# Formative Parental Competencies and Parenting Stress as Predictors of Children’s Self-Esteem

**DOI:** 10.11621/pir.2026.0203

**Published:** 2026-06-01

**Authors:** Claudia Pablos Cruz, Blanca Silvia Fraijo-Sing, César Octavio Tapia-Fonllem, Rosalba Valenzuela-Peñuñuri, Natalia Nieblas-Soto

**Affiliations:** a University of Sonora, Hermosillo, México

**Keywords:** positive parenting, formative parental competencies, parental stress, children’s self-esteem, structural equation model

## Abstract

**Background.:**

Nurturing and responsive parenting, along with parental competencies that foster child development, are the foundation for healthy emotional and psychological growth, as they provide children and adolescents with a secure environment in which they feel valued, understood, and capable of coping with everyday challenges. From this perspective, the quality of family interactions plays a central role in the development of children’s self-esteem.

**Objective.:**

To analyze the relationship among formative parental competencies, parental stress, and children’s self-esteem.

**Design.:**

The sample of 332 participants comprised 166 children aged 8 to 12 years and their caregivers. The E2P v.2 scale, the PSI-SF, and the Rosenberg Self-Esteem Scale were administered, after obtaining consent from the parents and/or caregivers and assent from the children.

**Results.:**

At a correlational level, formative parental competencies were negatively associated with parental stress, and positively associated with children’s self-esteem, while parental stress was negatively associated with that self-esteem. When examining the relationships using structural equation modeling, formative parental competencies were associated with less stress and higher self-esteem in children, without showing a direct effect of stress on the children’s self-esteem.

**Conclusion.:**

These findings suggest that the quality of parenting practices is associated with lower levels of parental stress and higher levels of children’s self-esteem, highlighting its potential role as a protective factor. This pattern of associations may inform the design of interventions aimed at strengthening parental competencies aligned with positive parenting, without implying causal effects.

## Introduction

Parenting and its association with children’s self-esteem are a leading focus of contemporary psychological research. Numerous studies agree that parenting practices exert a significant influence on a child’s self-esteem, understood as the positive or negative evaluation that individuals make of themselves (Rosenberg, 1965).

In particular, parental support and authoritative parenting styles, characterized by a combination of affection, open communication, and clear boundaries, promote children’s autonomy and responsibility within a structured yet nurturing environment. These styles are positively associated with higher levels of children’s self-esteem, while authoritarian styles are negatively related to these indicators. Furthermore, self-esteem is inversely linked to depressive symptoms, highlighting its protective function against emotional distress. Consistently, positive parenting practices are associated with a lower prevalence of depressive symptoms, while those based on psychological control are associated with an increase in these symptoms (Feldman, 2012; Pinquart & Nguyen, 2025).

Nurturing and responsive parenting forms the basis for healthy emotional and psychological development, providing children and adolescents with a safe environment where they feel valued, understood, and competent (Duarte et al., 2016). Such bonds foster self-confidence, emotional self-regulation, and the development of self-esteem. In contrast, rigid, authoritarian, or emotionally unsupportive parenting can weaken a child’s sense of self-worth, generate insecurity, and increase vulnerability to stress. In this sense, the quality of the parental bond is a key protective or risk factor for children’s and adolescents’ psychological well-being (Shen et al., 2025).

Positive parental competencies, understood as the set of practices that allow caregivers to respond flexibly and adaptively to the demands of raising children, represent a fundamental pillar for promoting the holistic development of children and adolescents (Bornstein, 2013; Gómez & Muñoz, 2015; Pinquart & Nguyen, 2025). The literature has consistently documented that sensitive families and structured parenting styles are associated with better outcomes in children’s well-being and self-esteem, while coercive or inconsistent practices are linked to less favorable results (Baumrind, 1991; Rodrigo et al., 2009; Shen et al., 2025). Parental competencies can be divided into four areas, according to Gómez and Muñoz (2015): a) bonding competencies; b) formative competencies; c) protective competencies; and d) reflective competencies.

Formative competencies refer to the set of knowledge, attitudes, and daily practices of parenting and child-rearing that are aimed at fostering development, learning, and socialization (Gómez & Muñoz, 2015). Thus, formative parental competencies are considered a foundation for children to explore the world and integrate social learning through modeling, dialogue, and interaction.

From a developmental perspective, promoting self-esteem in childhood plays a crucial role in preventing emotional problems and fostering healthy development, as it is linked to better physical and mental health, higher academic performance, and prosocial behaviors (Delval, 1998). Furthermore, self-esteem is a central component of psychological adjustment, reflecting the subjective assessment of one’s own personal worth and closely associated with socio-emotional functioning (Orth et al., 2018; Pinquart & Nguyen, 2025; Rosenberg, 1965).

On the other hand, the daily practice of parenting involves changing demands that require constant adaptation from caregivers (Ponnet et al., 2013). When these demands exceed available personal, social, or contextual resources, parental stress can emerge, defined as an adverse emotional response associated with the care and socialization of children (Wekerle et al., 2007). Empirical evidence indicates that high levels of parental stress can negatively affect children’s emotional development, increasing the likelihood of mental health problems in childhood (Tehrani et al., 2025). Furthermore, studies have shown that parental stress significantly relates to psychological well-being of the parents themselves, being associated with lower levels of life satisfaction and quality of life (Rusu et al., 2025), which is an issue that has become particularly relevant in contemporary discussions of family life.

Poor parental well-being is associated with less healthy parenting practices, characterized by less affection and less reciprocity in interactions with children, which are negatively related to parental competencies (Wekerle et al., 2007). These competencies have been identified as key determinants for the development of healthy self-esteem in children (Pinquart & Nguyen, 2025).

Although recent meta-analyses (*e.g.*, Pinquart & Gerke, 2025) have provided robust evidence on the associations between parenting practices and children’s psychological outcomes, several gaps remain. Less attention has been given to how these relationships operate within specific cultural contexts and developmental stages. In particular, middle childhood represents a critical period for the consolidation of self-esteem, yet it is often underrepresented or not explicitly differentiated in large-scale syntheses (González, et al., 2020; Oudhof & Robles, 2022). Parenting practices and their impact on child development are embedded within broader cultural contexts that shape beliefs, expectations, and interaction patterns within families. Cultural norms influence how parental competencies are expressed, how stress is experienced and managed, and how children interpret and internalize relational experiences. Therefore, examining these processes within specific sociocultural settings is essential for understanding both their universality and their contextual variability. This perspective highlights the importance of interpreting findings with consideration of the cultural milieu in which they are produced (Atzaba-Poria & Pike, 2015).

### Formative Parental Competencies and Parental Stress

Sahithya & Raman (2020) report that parenthood in a context that is itself stressful requires primary caregivers to develop positive resources that give them confidence and reduce the perceived stress in the exercise of parenthood when attending to various obligations in the same space-time.

The stress experienced by primary caregivers during child-rearing is called parental stress; and this has been defined in various studies (González Bernal & Guevara Marín, 2012; Hughes & Huth-Bocks, 2008; Padilla et al., 2014) as overwhelming negative feelings regarding parenting and anguish due to disapproving comments received in the context in which parenting is exercised.

According to Abidin (1995), negative feelings are based on caregivers’ self-evaluation of their resources for coping with parental demands. This author divides this self-evaluation into three dimensions: parental distress, which consists of each caregiver’s perception of their parenting; dysfunctional parent–child interaction, which involves caregivers’ assessment of their expectations of their children and the feedback they receive from them; and finally, a difficult child, which encompasses the child’s psychological attributes and behaviors that create difficulties for caregivers in raising their children (Faber & Mazlish, 2014). In this approach, Rodrigo et al. (2009) explain that the degree of satisfaction with being a parent can positively or negatively influence the emotional health, self-esteem, and well-being of children. Therefore, the competencies developed by primary caregivers are considered crucial in the development of the family unit and the development of children (Martín et al., 2013).

Strengthening parental competencies can contribute to reducing parental stress and increasing the perception of competence in caregivers, as studies have shown from programs and interventions that low levels of parental stress give a feeling of competence, which is reflected in behavioral changes in children (Sahithya et al., 2020; Pisterman et al., 1992).

### Formative Parental Competencies, Parental Stress, and Their Relationship with Children’s Self-Esteem

Positive parenting and children’s self-esteem have been studied through various interventions, which have found that positive parenting has a direct and positive effect on children’s self-esteem (Batool, 2020). Sugiarti et al. (2021) reported that positive parenting is significantly associated with self-esteem in children, from infancy to adolescence, highlighting that dialogue, understanding, and the maintenance of close relationships by parents contribute to children valuing themselves more and perceiving themselves as more competent. Thus, self-esteem functions as a key protective factor in the emotional development of children and adolescents, strengthening their ability to cope with stress and reducing emotional vulnerability (Peng et al., 2021). On the other hand, numerous studies have analyzed self-esteem as a predictor of behavior and psychological adjustment (Leary & MacDonald, 2005), highlighting that high levels of self-esteem are associated with adaptive behaviors and greater wellbeing (Diener, 1984; Okada, 2010; Taylor & Brown, 1988).

While various risk factors have been associated with low self-esteem in children, evidence suggests that inconsistent parenting is one of the most significant factors (Brown et al., 1990; Rosenberg et al., 1989). Studies have shown that parental characteristics such as maternal emotional stability significantly influence children’s mental health (Hoghughi & Long, 2004), while parental stress, depression, and low self-esteem in caregivers are associated with poorer attachment quality and reduced parental efficacy (Leerkes & Burney, 2007).

The literature has also demonstrated a close relationship between parental stress, parenting, and children’s self-esteem. Several studies have recorded a significant association between parental depression and parenting styles characterized by low care and high control (McGinn et al., 2005; Mezulis et al., 2006). Within this context, stress management programs based on the Lazarus & Folkman (1984) coping model have shown that reducing parental stress can indirectly contribute to decreasing depressive symptoms in children (Ajilchi et al., 2013).

Furthermore, parental stress is linked negatively to children’s self-esteem, potentially leading them to unfavorable interpretations of reality and increasing anxiety, self-criticism, and feelings of inadequacy (Pappa et al., 2024). Self-esteem, as a central component of the self-concept, begins to develop in the early stages of life, a process in which the family plays a fundamental role as the first social and educational environment children experience (Delval, 1998; Lodi-Smith & DeMarree, 2018). From this perspective, daily interactions with parents are crucial for the development of self-concept and self-esteem, directly influencing how children perceive and value themselves (Curran et al., 2018). Positive parenting practices foster the development of a healthy self-concept and, consequently, positive self-esteem, while inadequate practices can generate negative labels that become the basis of a dysfunctional self-concept and low self-esteem (Rohmalimna et al., 2022). As a result, parental stress emerges as a relevant factor that can interfere with the quality of parenting and, indirectly, with children’s self-esteem (Moreira et al., 2015).

**Figure 1.**
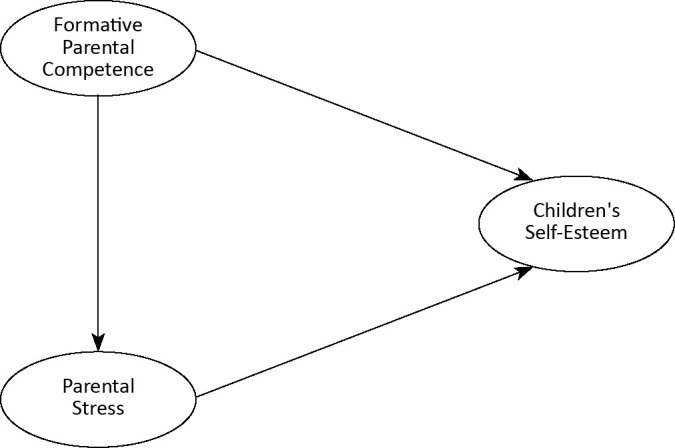
Hypothetical model of the study

Following this logic, the present study aims to determine the relationship between formative parental competencies and some dimensions of parental stress, as well as its connection to children’s self-esteem. The goal is to provide evidence to deepen the understanding of risk and protective factors in the family environment, contribute to understanding the challenges confronting today’s families, and highlight the importance of designing interventions aimed at families to promote healthy child development (Baumrind, 1991; Gómez & Muñoz, 2015; Rodrigo et al., 2009; Rosenberg, 1965; Ryff, 2014). Self-esteem was selected as the primary child outcome due to its central role as an integrative indicator of socio-emotional development. Unlike more transient emotional states such as stress or affect, self-esteem reflects a relatively stable evaluation of the self, shaped by cumulative relational experiences, particularly within the family. In childhood, self-esteem is closely associated with emotional well-being, stress perception, and self-regulatory processes (Krauss et al., 2020), making it a particular indicator of psychological adjustment. Given the aim of this study to examine the developmental implications of formative parental competencies, focusing on self-esteem allows for the assessment of a broader and more enduring psychological outcome. Although parental characteristics, including parental self-esteem, may indirectly influence children’s self-views through modeling and family dynamics, previous evidence suggests that theses influences are often mediated by parenting behavior and efficacy beliefs rather than directly transmitted (Fang et al., 2021). Therefore, the present study focused specifically on children’s self-esteem in order to capture their subjective self-evaluations more directly. Although other variables such as affect or stress may also be relevant, their inclusion would have extended the model beyond its intended scope. **Figure 1** presents the following hypotheses:

*H_1_*: Formative parental competencies are negatively related to parental stress.

*H_2_*: Formative parental competencies are positively related to children’s self-esteem.

*H_3_*: Parental stress is negatively related to children’s self-esteem.

In the study of parenting and child development, multiple emotional and relational variables have been identified as relevant for understanding children’s psychological adjustment. Among these, affective dimensions such as positive and negative affect have shown significant associations with well-being outcomes (Huang et al., 2026). However, the present study focuses specifically on some dimensions of parental stress as a central process variable within the parenting framework. Parental stress has been consistently conceptualized as a proximal mechanism that directly influences parenting practices, particularly by reducing responsiveness and increasing the likelihood of dysfunctional interactions. From this perspective, stress is not only an indicator of parental well-being, but also a key factor shaping the quality of the parent–child relationship. In contrast, affective dimensions are often considered broader emotional states or dispositional characteristics that, while relevant, operate at a different level of analysis. Given the aim of this study to examine modifiable parenting-related processes linked to formative parental competencies, some dimensions of parental stress were selected as a theoretically grounded and intervention-relevant variable.

## Methods

### Participants

The study sample consisted of 332 participants: 166 adults (*M =* 37.7, *SD =* 6.7) and their children, aged 8 to 12 years (*M =* 10.06, *SD =* 1.18), enrolled in 13 public elementary schools in the northern, southern, central, and western areas of Hermosillo, Mexico. Regarding their relationship to the children, the informants were mothers (86.7%), fathers (4.8%), grandmothers (6.0%), grandfathers (.6%), and other relatives (1.8%). All participants were invited and voluntarily agreed to participate in the study in person. The sample size is consistent with commonly recommended guidelines for structural equation modeling, which suggest a minimum ratio of 5 to 10 observations per estimated parameter and sample sizes of approximately 150 to 200 participants for models of moderate complexity (*e.g.*, Hair et al., 2019; Kline, 2016).

### Procedure

Initially, modifications were made to the instruments following international recommendations for the cross-cultural adaptation of psychometric instruments, which emphasize the importance of maintaining semantic, idiomatic, and conceptual equivalence (Beaton et al., 2000; Muñiz et al., 2013); The language and wording of the items were adjusted in the positive parenting scale and parental stress index, with the aim of making them understandable and relevant to the sociocultural context of the participants, while maintaining the conceptual equivalence of the original version of the instruments (Hambleton et al., 2005).

Subsequently, mothers, fathers, or family members who served as primary caregivers for children between the ages of 8 and 12, attending 13 public elementary schools in Hermosillo, Mexico, were invited to participate. Prior permission was obtained from both the principals of the participating schools and the staff of the Unit of Support Services for Regular Education (USAER) to convene the interested parties. The questionnaires were administered from February to June 2025. Families were scheduled for specific days, previously agreed upon with each school, to complete the questionnaires in person using paper-and-pencil format. The process took approximately 25 to 30 minutes for the caregivers.

Later that same day, the children were asked to complete the questionnaires, which took 10 to 15 minutes. Before administering the questionnaires, both the caregivers and the children signed the corresponding informed consent forms. The information obtained was handled confidentially; the responses were coded to guarantee the anonymity of the participants, and subsequently the data was entered into electronic databases for statistical analysis.

### Data Analysis

Using the Statistical Package for the Social Sciences (SPSS v25.0), the psychometric properties and descriptive statistics of the scales were analyzed. In the first stage, an Exploratory Factor Analysis (EFA) was conducted to identify the underlying structure of the items in each instrument, helping to reduce the number of indicators of the latent variables by eliminating values with a loading lower than .30 (Hoyle & Duvall, 2004; Yong & Pearce, 2013). Subsequently, to estimate the internal consistency of the scales, a Cronbach’s alpha analysis was performed (Swerdlik & Cohen, 2006), considering scores above .60 as acceptable (Nunnally & Bernstein, 1994).

Following this, Confirmatory Factor Analyses (CFA) were performed using EQS 6.1 software for each scale to determine construct validity. The strength of association between variables was also calculated using Pearson’s correlation coefficient (Triola, 2009). Finally, to test the hypotheses, Structural Equation Modeling (SEM) was used to determine the relationship between variables, employing various statistical measures to estimate the model’s goodness of fit (Kline, 2016; Ruiz et al., 2010). Values above .90 were considered indicative of acceptable model fit for the Normed Fit Index (NFI), Non-Normed Fit Index (NNFI), and Comparative Fit Index (CFI). For the Root Mean Square Error of Approximation (RMSEA), values below .08 were considered indicative of acceptable fit, whereas values below .05 suggested a good model fit (Bentler & Bonett, 1980; Chou & Bentler, 1993; Smith & McMillan, 2001).

### Questionnaires

Instruments were administered to the parents and the children separately. The scales used are as follows:

*Positive Parenting Scale (E2P v.2)* (Gómez & Contreras, 2019). A scale that evaluates parental competencies from the perspective of positive parenting, consisting of four factors: a) bonding parental competencies, referring to actions aimed at promoting a secure attachment style and adequate socio-emotional development; b) formative competencies, which are actions aimed at promoting development, learning, and socialization; c) protective competencies, which are behaviors that favor the physical and emotional integrity of children; d) reflective competencies, which refer to one’s own parenting and the monitoring of current parenting practices. It consists of 56 items and uses a 5-point Likert scale response format (1 = never, 5 = always). The original scale has psychometric validity (α = .94). The E2P v.2 scale was originally proposed as a four-factor instrument. However, for the purposes of the present study, only the Formative Parental Competencies (FPC) factor was included due to its theoretical relevance to the proposed model and its suitability for examining parenting practices within the sociocultural context of the participating families.

*Parenting Stress Index-4 Short Form (PSI-SF).* A scale that measures parental stress (Abidin, 1995; Díaz-Herrero et al., 2010). Its abbreviated form consists of 36 items on a 5-point Likert scale and allows for the rapid identification of parents with high stress levels stemming from both personal characteristics and their children’s behavioral traits. Originally, the PSI-SF was structured into three subscales: a) Parental Distress; b) Dysfunctional Parent-Child Interaction; and c) Difficult Child, which together provide an overall Total Stress Index (α = .91). For the purposes of the present study, only Parental Distress (PD) and Difficult Child (DC) subscales were included, as these dimensions were considered most directly related to the experience of parental stress examined in the proposed model.

*Rosenberg Self-Esteem Scale* (Rosenberg, 1965). Consists of 10 Likert-type items, five written in a positive way and five in a negative way, which assess the person’s general attitude towards himself, his worth and personal acceptance (Atienza et al., 2000; Fleming & Courtney, 1984; McCarthy & Hoge, 1982; Wylie, 1974) (α = .88). Seen in *Table 1* as SE.

## Results

*Table 1* shows the correlations of the study variables, as well as the internal consistency values and descriptive statistics. The results for calculating internal consistency using Cronbach’s alpha coefficient were satisfactory, as all scales showed values above .70, with the Self-Esteem variable having the lowest (α = .76). Regarding the distribution of the data, skewness and kurtosis values indicate that, overall, the variables exhibit moderate deviations from normality; in particular, the variable Formative Parental Competencies showed pronounced negative skewness (-2.00) and elevated kurtosis (4.75), suggesting a concentration of responses at the higher end of the scale. This distributional pattern may reduce variability and slightly attenuate the magnitude of associations with other variables. In contrast, the remaining variables presented skewness and kurtosis values within acceptable ranges, supporting the adequacy of the data for subsequent analyses. Given that all values fell within commonly accepted thresholds, these deviations were not considered severe enough to compromise the validity of the analyses.

**Table 1:** Univariate Statistics and Correlations of the Study

	Mean	SD	Skewness	Kurtosis	Alpha	FPC	PS	DC	PD	SE
**FPC**	4.62	.51	–2.00	4.749	.945	1				
**PS**	2.12	.69	.46	–.041	.925	–.388**	1			
**DC**	2.08	.75	.54	–.285	.851	–.254**	.822*	1		
**PD**	2.33	.96	.62	–.220	.802	–.246**	.766**	.356**		
**SE**	2.79	.91	–.38	–.989	.760	.199**	–.174**	–.095	–.170*	1

Note. N = 166; FPC = Formative Parental Competencies. PS = Parental Stress. DC = Difficult Child. PD = Parental Distress. SE = Self-esteem. Pearson *p < .05. **p < .001.

Based on the Pearson correlation results, a pattern of associations consistent with the proposed theoretical model was observed. Formative parental competencies were found to be negatively and significantly related to Parental Stress (*r* = −.388, *p <* .001), indicating that higher levels of formative parental competencies are associated with lower levels of stress in raising children. Similarly, formative parental competencies showed a positive and significant correlation with Children’s Self-Esteem (*r* = .199, *p <* .001). This relationship, although of low magnitude, suggests that greater formative competence—that is, higher levels of guidance, explanation of rules, and reflective support from caregivers—is associated with lower levels of negative self-thought in children, reflecting better self-esteem. Consistent with this finding, Parental Stress was negatively and significantly associated with Children’s Self-Esteem (*r* = −.174, *p <* .001), suggesting that higher levels of stress in parents tend to be related to lower levels of self-esteem in children. Taken together, these associations support the relevance of formative parental competencies as a protective factor both for reducing parental stress and for promoting better self-esteem in children.

Confirmatory Factor Analysis (CFA) was performed using the robust maximum likelihood estimation method to obtain goodness-of-fit indices and factor loadings for each of the indicators corresponding to the study variables (Stommel et al., 1992). The measurement models showed a satisfactory fit to the data, apart from the second order construct dysfunctional interaction, belonging to parental stress, which showed factor loadings below .30. Therefore, this factor was removed from subsequent analyses. The remaining latent variables and their respective factor loadings were statistically significant, indicating that the constructs were adequately represented by their indicators.

**Figure 2:**
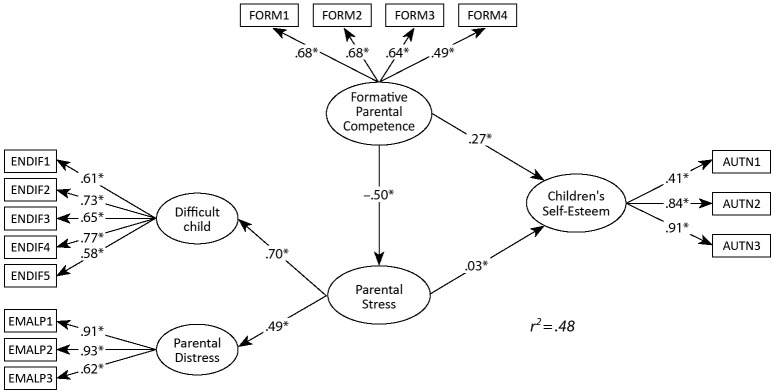
Structural model of the relationship among Formative Parental Competence, Parental Stress, and Children’s Self-Esteem, with their standardized estimates

*Note. Goodness of fit: χ^2^(95) = 132.03, χ^2^/df = 1.39, CFI=.97, TLI (BBNNFI)= .96, RMSEA = .04 (90% CI [.026, .067]), SRMR = .060.*

In the structural model, the relationship between formative parental competencies, parental stress, and child self-esteem was examined (**Figure 2**). Formative parental competencies showed a significant negative effect on parental stress (β = –.50), a construct consisting of the perception of a difficult child and parental distress, indicating that greater parenting competencies are associated with lower stress levels. Regarding child self-esteem, formative parental competencies had a positive influence (β = .27), demonstrating their role as a relevant protective factor in the child’s emotional development, fostering a more positive self-assessment.

The direct effect of parental stress on self-esteem, although positive, was practically null (β = .03), indicating that once formative parental competencies are considered within the model, parental stress does not exert a significant or relevant influence on children’s self-esteem. The effect of parental stress on children’s self-esteem is not independent, but rather conditioned by positive formative parental resources. The model explained 48% of the variance in children’s self-esteem (*r*^2^= .48) and showed adequate goodness-of-fit indices as seen in **Figure 1**, which is consistent with the theoretical coherence of the model and suggests a pattern of associations whereby formative parental competencies are linked to lower parental stress and to higher levels of children’s self-esteem, without implying causal direction.

The structural model showed that several of the hypothesized relationships were statistically supported (*Table 2*). Specifically, Formative Parental Competencies exhibited a significant negative association with Parental Stress (β = –.534, SE = .082, z = –3.015, *p <* .01), indicating that higher levels of these competencies are linked to lower levels of Parental Stress. In turn, Parental Stress was positively associated with both Difficult Child (β = .701, SE = .285, z = 3.491, *p <* .001) and Parental Distress. Although the latter effect was of moderate magnitude (β = .496, SE = .296), its associated variability resulted in a wider confidence interval, suggesting a more cautious interpretation of this relationship.

**Table 2:** Standardized Path Coefficients of the Structural Model

Path	β	SE	Z	*p*	95% CI
Formative Stress Parental Competencies → Parental	–.503	.082	–3.015	< .01	[–.69, –.37]
Parental Stress → Difficult Child	.701	.285	3.491	< .001	[ .11, 1.22]
Parental Stress → Parental Distress	.496	.296	3.565	< .001	[–.10, 1.07]
Formative self-esteem Parental Competencies → Children’s	.273	.141	1.918	.055	[–.01, .54]
Parental Stress → Children’s self-esteem	.006	.356	.037	ns	[–.69, .70]

*Note. β = standardized regression coefficient. SE = standard error. z = test statistic (estimate divided by its standard error). p = significance level. CI = 95% confidence interval. Structural paths represent hypothesized directional relationships between latent constructs. Coefficients are considered statistically significant at p < .05.*

Regarding children’s self-esteem, the direct effect of Parental Stress was negligible (β = .006, SE = .356, z = .037, *p* > .05). Similarly, Formative Parental Competencies showed a positive association with children’s self-esteem that approached, but did not reach, conventional levels of statistical significance (β = .273, SE = .141, z = 1.918, *p* = .055), indicating a potential trend worthy of further exploration.

## Discussion

This study aimed to analyze the relationship among formative parental competencies, parental stress, and children’s self-esteem in a sample of families with school-aged children in northwestern Mexico. Formative parental competencies were defined as the ability of parents to organize a teaching and learning environment aligned with positive parenting, which fosters the development of competencies in children that allow them to function independently and adaptively in society (Barudy & Dantagnan, 2005; Gómez & Contreras, 2019). Parental stress was understood as the negative emotional responses of parents to the demands of caring for and socializing their children (Abidin, 1995; Ponnet et al., 2013; Wekerle et al., 2007). Self-esteem was defined as the overall evaluation a person makes of their own worth (Atienza et al., 2000; Rosenberg, 1965).

Several studies have already demonstrated that parental stress is a significant risk factor for the successful implementation of positive parenting practices, as high stress levels are associated with reduced sensitivity and responsiveness of caregivers to their children’s needs (Martorell & Bugental, 2006; Rodriguez-Jenkins & Marcenko, 2014). This has been consistently linked to impairments in children’s socio-emotional development (Pinquart & Gerke, 2019; Shen et al., 2025). The results of the present study provide empirical evidence that reinforces the importance of formative parental practices aligned with positive parenting as a key protective factor in children’s socio-emotional development, particularly in building and strengthening self-esteem. Also, these findings contribute to current discussion on family functioning and highlight the relevance of parental competencies to promote healthy family environments (Angley et al., 2015).

The psychometric analyses showed adequate levels of internal consistency and construct validity in the scales used, confirming the appropriateness of the adaptation for use with the Mexican population. However, it is important to mention that the omission of the dysfunctional interaction factor within the parental stress construct in the structural model analysis suggests the need for further in-depth evaluation of this dimension.

Regarding the correlational analyses, a pattern of associations consistent with the theoretical framework and previous research was identified, which has shown that the authoritative parenting style, which shares fundamental characteristics with positive parental competencies, maintains a direct and significant relationship with children’s self-esteem (Firouzkouhi Moghaddam et al., 2017; Pinquart & Gerke, 2019). In addition, in the present study, it was observed that formative parental competencies, aligned with positive parenting, were positively and significantly related to children’s self-esteem, indicating that practices such as clear guidance, reflective accompaniment, and the explanation of rules favor a more positive self-evaluation in children (Marsh, 1996). Likewise, as in other studies where it has been found that parents with a greater sense of parental competence have lower levels of parental stress (Dincer & Tunç, 2023; Molano et al., 2023), in the present study, formative parental competencies were negatively and significantly associated with parental stress, suggesting that greater formative resources aligned with positive parenting in raising children are linked to lower levels of parental distress and perception of difficulty in the parental exercise. It has been found that higher levels of parental stress are associated with more negative perceptions of parental behavior by their children and, through the parenting practices implemented, are related to lower self-esteem in children (Put-nick et al., 2008). On the other hand, it is important to mention that some research found no significant relationship between parental stress and children’s self-esteem, suggesting that in some contexts, the relationship between parental stress and children’s self-esteem is not always direct or significant (Prasana & Sam, 2024).

In the present study, the correlational analysis showed that parental stress had a negative and significant relationship with children’s self-esteem, demonstrating that high levels of stress in caregivers tend to be associated with lower self-esteem in their children. These findings are consistent with previous studies that indicate that higher levels of family functioning are associated with better child well-being (Gaspar et al., 2022).

Furthermore, in the structural equation model, which allowed for a deeper understanding of the relationships between variables by analyzing direct effects, the results showed that formative parental competencies have a significant effect on both reducing parental stress and strengthening children’s self-esteem. In contrast, parental stress did not show a significant effect on self-esteem once parental competencies were incorporated into the model, suggesting that the association between parental stress and children’s socio-emotional development does not occur in isolation, but rather is linked to the quality of parenting practices. This finding may not reflect the multifactorial nature of children’s self-esteem, which is influenced not only by family dynamics but also by broader developmental and social contexts. These results reinforce the idea that the level of stress itself is not the main determinant of children’s emotional adjustment, but rather the caregivers’ ability to implement formative parental resources even in demanding or pressured contexts. Taken together, these findings suggest that formative parental competencies may act as a protective mechanism that buffers the potential negative effects of parental stress on children’s self-esteem (Delvecchio et al., 2020). This interpretation is consistent with theoretical models of parenting that emphasize the role of parental resources in shaping family functioning and reducing vulnerability to stress in caregiving contexts (Belsky, 1984). At the same time, these findings highlight the importance of considering additional individual and relational variables that may further explain children’s psychological adjustment.

Future research should consider including parental self-esteem, as parental self-related beliefs and efficacy perceptions may indirectly shape children’s self-evaluations through modeling processes, parenting behaviors, and family dynamics (Busquets et al., 2022; Niermann et al., 2022). Examining both parent and child self-esteem could therefore provide a more comprehensive understanding of intergenerational influences on psychological adjustment, particularly from a dyadic perspective that captures reciprocal family processes.

In addition, although affective variables were not included in the present study, incorporating both positive and negative affect may contribute to a more comprehensive understanding of the observed relationships. Positive affect has been consistently associated with lower perceived stress and higher self-esteem and self-efficacy, as it promotes adaptive coping, cognitive flexibility, and more favorable self-evaluations. In contrast, negative affect is typically linked to higher stress and diminished self-related beliefs, as it is associated with maladaptive cognitive patterns such as rumination and threat appraisal (Aktu, 2024). As suggested by Moè and Katz (2018), affective experiences play a central role in shaping individuals’ perceptions of competence and their emotional adjustment in achievement contexts, reinforcing the relevance of considering these dimensions in future studies.

These findings also have important educational and practical implications. Interventions aimed at strengthening formative parental competencies may contribute to promoting children’s self-esteem while also mitigating the impact of parental stress. Such interventions should be culturally sensitive and consider values, beliefs, and contextual conditions of the families involved. Additionally, given the developmental stage of the children included in this study, educational programs may benefit from incorporating strategies that promote supportive parent–child interactions during middle childhood. Adapting these interventions to different cultural and developmental contexts may further enhance their relevance and effectiveness (Jeong et al., 2021).

Overall, these findings should be interpreted in terms of associations rather than causal effects. Although the model was theoretically specified with directional paths, the cross-sectional nature of the data does not allow for establishing temporal or causal relationships among variables.

## Conclusion

The proposed model explained a considerable proportion of the variance in children’s self-esteem, which supports its theoretical and empirical consistency (Firouzkouhi Moghaddam et al., 2017; Pinquart & Gerke, 2019). In particular, the associations between formative parental competencies and children’s self-esteem, as well as between parental stress and children’s self-esteem, underscore the central role of positive formative parenting practices as a relevant explanatory factor in children’s socio-emotional development, beyond the independent contribution of some dimensions of parental stress (Orth & Robins, 2014).

The findings of this study are consistent with previous research highlighting the protective role of positive parenting practices in children’s socio-emotional development (Lansford et al., 2014; Morris et al., 2007; Pinquart, 2017). However, these results should be interpreted within the specific cultural and developmental context of the study. Parenting practices and experiences of parental stress may vary across cultural settings due to differences in social norms, family structures, and contextual stressors (Bornstein, 2017; Lansford et al., 2021). Likewise, the age group examined in this study (children aged 8 to 12 years) represents a developmental stage in which self-esteem is particularly sensitive to family interactions, which may limit the generalizability of the finding to younger children or adolescents (Orth & Robins, 2014).

Overall, the present findings contribute to the literature by providing evidence from a specific cultural and developmental context, while also underscoring the need for further cross-cultural and longitudinal research to examine the stability and variability of these relationships over time.

## Limitations

Finally, although the results provide solid evidence, the main limitation of this study is the sample size, which may restrict the generalizability of the findings. However, the inclusion of participants from different geographic areas of the city contributes to the internal diversity of the sample (Tipton, 2017). It is therefore necessary to replicate the proposed model in larger samples and across diverse sociocultural contexts. Nevertheless, the present findings constitute a relevant contribution to research on positive parenting in Mexican contexts and provide empirical support for the design of interventions aimed at strengthening family well-being and children’s socio-emotional development through positive parenting practices.

Several methodological limitations should also be considered when interpreting the findings. First, the study focused exclusively on parental stress as the primary emotional variable. Although this decision was theoretically grounded, the inclusion of additional emotional dimensions, such as positive and negative affect, could provide a more comprehensive understanding of family emotional dynamics.

Limitations related to measurement should likewise be acknowledged. Although the Rosenberg Self-Esteem Scale (RSES) is a widely used and well-validated instrument, it was originally developed for adolescents and adults. Therefore, its application to children aged 8 to 12 may present challenges related to developmental appropriateness and item comprehension. While efforts were made to ensure that participants understood the items, future research would benefit from employing self-esteem measures specifically designed or validated for children to obtain more developmentally sensitive assessments. In addition, although the E2P v.2 scale was originally developed as a multidimensional measure, the present study focused exclusively on the dimension related to formative parental competencies. While this allowed for a more specific examination of the construct, it may limit comparability with studies using the full instrument. Future research should consider employing the complete scale to capture a broader range of parental competencies.

Other limitations relate to the characteristics of the sample and the analytical approach. Subgroup analyses (*e.g.*, by child gender or caregiver type) were not conducted, as the primary aim was to test a general model. However, this limits the ability to identify potential differences in the relationships examined. Future studies could address this issue through multi-group structural equation modeling. The caregiver sample was predominantly composed of mothers (86.7%), meaning that the findings primarily reflect maternal parenting practices and experiences and may not be fully generalizable to fathers or other caregivers. Finally, because the study was conducted within a specific sociocultural context, the findings may not be generalized to populations with different cultural backgrounds, family structures, or socioeconomic conditions. Replication across diverse cultural settings would therefore strengthen the external validity of the proposed model.

## References

[ref1] Abidin, R.R. (1995). Parenting Stress Index (PSI) manual (3rd ed.). Pediatric Psychology Press.

[ref2] Ajilchi, B., Kargar, F.R., & Ghoreishi, M.K. (2013). Relationship between the parenting styles of overstressed mothers with their children’s self-esteem. Procedia-Social and Behavioral Sciences, 82, 496–501. 10.1016/j.sbspro.2013.06.299

[ref3] Aktu, Y. (2024). The role of parenting stress on parenting self-efficacy and parental burnout among Turkish parents: A moderated mediation model. BMC Psychology, 12, 475. 10.1186/s40359-024-01980-7 39252138 PMC11386338

[ref4] Angley, M., Divney, A., Magriples, U., Kershaw, T. (2015). Social support, family functioning and parenting competence in adolescent parents. Maternal and Child Health Journal, 19, 67–73. 10.1007/s10995-014-1496-x 24833286 PMC4233010

[ref5] Atienza, F.L., Moreno, Y., & Balaguer, I. (2000). Análisis de la dimensionalidad de la Escala de Autoes-tima de Rosenberg en una muestra de adolescentes valencianos [Analysis of the dimensionality of the Rosenberg Self-Esteem Scale in a sample of Valencian adolescents]. Revista de Psicología Universitas Tarraconensis [Journal of Psychology Universitas Tarraconensis], 22, 29–42.

[ref6] Atzaba-Poria, N., & Pike, A. (2015). Through a cultural lens: Links between maternal and paternal negativity and children’s self-esteem. Journal of Cross-Cultural Psychology, 46(5), 702–712. 10.1177/0022022115581011

[ref7] Barudy, J., Dantagnan, M. (2005). Los buenos tratos a la infancia: parentalidad, apego y resilencia [Good treatment of children: parenting, attachment and resilience]. Gedisa.

[ref8] Batool, S.S. (2020). Academic achievement: Interplay of positive parenting, self-esteem, and academic procrastination. Australian Journal of Psychology, 72(2), 174–187. 10.1111/ajpy.12280

[ref9] Baumrind, D. (1991). Effective parenting during the early adolescent transition. In P.A. Cowan & E.M. Hetherington (Eds.), Family transitions: Advances in family research series (pp. 111–163). Lawrence Erlbaum Associates.

[ref10] Beaton, D.E., Bombardier, C., Guillemin, F., & Ferraz, M.B. (2000). Guidelines for the process of cross-cultural adaptation of self-report measures. Spine, 25(24), 3186–3191. 10.1097/00007632-200012150-00014 11124735

[ref11] Belsky, J. (1984). The Determinants of Parenting: A Process Model. Child Development, 55(1), 83–96. 10.2307/1129836 6705636

[ref12] Bentler, P.M., Bonett, D.G. (1980). Significance tests and goodness of fit in the analysis of covariance structures. Psychological Bulletin, 88(3), 588–606. 10.1037/0033-2909.88.3.588

[ref13] Bornstein, M.H. (2013). Parenting and child mental health: A cross-cultural perspective. World Psychiatry, 12(3), 258–265. 10.1002/wps.20071 24096792 PMC3799257

[ref14] Bornstein, M.H. (2017). The Specificity Principle in Acculturation Science. Perspectives on Psychological Science: a Journal of the Association for Psychological Science, 12(1), 3–45. 10.1177/1745691616655997 28073331 PMC5234695

[ref15] Brown, G.W., Bifulco, A., Veiel, H.O.F., & Andrews, B. (1990). Self-esteem and depression. Social Psychiatry and Psychiatric Epidemiology, 25, 225–234. 10.1007/BF00788643 2237603

[ref16] Busquets, M., Glatz, T., Kiang, L., & Buchanan, C. (2022). Child-invested contingent self-esteem and parenting: Exploring differentiations by child successes or failures and ethnicity/race. Journal of Social and Personal Relationships, 39 (6), 1669–1692. 10.1177/02654075211062073

[ref17] Chou, C.P., & Bentler, P.M. (1993). Invariant standardized estimated parameter change for model modification in covariance structure analysis. Multivariate Behavioral Research, 28(1), 97–110. 10.1207/s15327906mbr2801_6 26824997

[ref18] Curran, J.A., Bishop, A., Chorney, J., MacEachern, L., & Mackay, R. (2018). Partnering with parents to advance child health research. Healthcare Management Forum, 31(2), 45–50. 10.1177/0840470417748442 29400092

[ref19] Delval, J. (1998). El desarrollo humano [Human development]. Siglo XXI Editores.

[ref20] Delvecchio, E., Germani, A., Raspa, V., Lis, A., & Mazzeschi, C. (2020). Parenting styles and child’s wellbeing: The mediating role of the perceived parental stress. Europe’s Journal of Psychology, 16 (3), e2013. 10.5964/ejop.v16i3.2013 PMC7909500 33680196

[ref21] Díaz-Herrero, Á., Brito de la Nuez, A.G., López Pina, J.A., Pérez-López, J., & Martínez-Fuentes, M.T. (2010). Estructura factorial y consistencia interna de la versión española del Parenting Stress Index–Short Form [Factor structure and internal consistency of the Spanish version of the Parenting Stress Index-Short Form]. Psicothema, 22(4), 1033–1038. https://www.psicothema.com/pdf/3801.pdf 21044549

[ref22] Diener, E. (1984). Subjective well-being. Psychological Bulletin, 95(3), 542–575. 10.1037/0033-2909.95.3.542 6399758

[ref23] Dincer, D., & Tunç, E.B. (2023). Parental competence, parenting stress, family harmony, and perceived available support among mothers with children aged 3–6 years. Research on Education and Psychology, 7(3), 15–41. 10.54535/rep.1353500

[ref24] Duarte, R.L., García, R.N., Rodríguez, C.E., & Bermúdez, J.M.E. (2016). Las prácticas de crianza y su relación con el vínculo afectivo [Parenting practices and their relationship with the emotional bond]. Revista Iberoamericana de Psicología: Ciencia y Tecnología, 9(2), 113–124. https://dialnet.unirioja.es/metricas/documentos/ARTREV/6124351

[ref25] Faber, A., & Mazlish, E. (2014). Cómo hablar para que los niños escuchen y como escuchar para que los niños hablen [How to talk so that children will listen and how to listen so that children will talk.]. Diana.

[ref26] Fang, Y., Boelens, M., Windhorst, D.A., Raat, H., & van Grieken, A. (2021). Factors associated with parenting self-efficacy: A systematic review. Journal of Advanced Nursing, 77, 2641–2661. 10.1111/jan.14767 33590585 PMC8248335

[ref27] Feldman, R. (2012). Parent–infant synchrony: A biobehavioral model of mutual influences in the formation of affiliative bonds. Monographs of the Society for Research in Child Development, 77(2), 42–51. 10.1111/j.1540-5834.2011.00660.x

[ref28] Firouzkouhi Moghaddam, M., Validad, A., Rakhshani, T., & Assareh, M. (2017). Child self-esteem and different parenting styles of mothers: A cross-sectional study. Archives of Psychiatry and Psychotherapy, 19(1), 37–42. 10.12740/APP/68160

[ref29] Fleming, J.S., & Courtney, B.E. (1984). The dimensionality of self-esteem: II. Hierarchical facet model for revised measurement scales. Journal of Personality and Social Psychology, 46(2), 404–421. 10.1037/0022-3514.46.2.404

[ref30] Gaspar, T., Gomez-Baya, D., Trindade, J.S., Botelho Guedes, F., Cerqueira, A., & de Matos, M.G. (2022). Relationship between family functioning, parents’ psychosocial factors, and children’s well-being. Journal of Family Issues, 43(9), 2380–2397. 10.1177/0192513X211030722

[ref31] Gómez, E., & Muñoz, M. (2015). Escala de Parentalidad Positiva E2P. Manual [E2P Positive Parenting Scale. Manual]. Fundación Ideas para la Infancia.

[ref32] Gómez, E., & Contreras, L. (2019). Escala de Parentalidad Positiva – E2P v.2: Manual [Positive Parenting Scale - E2P v.2: Manual]. Fundación América por la Infancia.

[ref33] González Bernal, M.R., & Guevara Marín , I.P. (2012). Estrés parental, trato rudo y monitoreo como factores asociados a la conducta agresiva [Parental stress, rough treatment and monitoring as factors associated with aggressive behavior]. Universitas Psychologica1 [University of Psychology], 11(1), 241–254. 10.11144/Javeriana.upsy11-1.eptr

[ref34] González, C.M.F., Guevara-Benítez, Y., Vargas, N.F., Valencia, A.I.O., & García, R.C. (2020). Habilidades sociales y autoestima en niños escolarizados mexicanos: Su relación con prácticas parentales [Social skills and self-esteem in Mexican schoolchildren: Their relationship with parental practices]. European Scientific Journal, 16 (34), 144. 10.19044/esj.2020.v16n34p144

[ref35] Hambleton, R.K., Merenda, P.F., & Spielberger, C.D. (2005). Issues, designs, and technical guidelines for adapting tests into multiple languages and cultures. In R.K. Hambleton, P.F. Merenda, & C.D. Spiel-berger (Eds.), Adapting educational and psychological tests for cross-cultural assessment (pp. 3–38). Lawrence Erlbaum Associates. 10.4324/9781410611758

[ref36] Hoghughi, M.S., & Long, N. (Eds.). (2004).Handbook of parenting: Theory and research for practice. Sage. 10.4135/9781848608160

[ref37] Hoyle, R.H., & Duvall, J.L. (2004). Determining the number of factors in exploratory and confirmatory factor analysis. In D. Kaplan (Ed.), The SAGE handbook of quantitative methodology for the social sciences (pp. 301–315). Sage. 10.4135/9781412986311.n16

[ref38] Huang, L., Wu, W., & Yang, F. (2026). Parenting style and subjective well-being in children and youth: A meta-analysis. PsychologicalReports, 129(2), 927–946. 10.1177/00332941241256883 38772039

[ref39] Huth-Bocks, A.C., Hughes, H.M. (2008). Parenting stress, parenting behavior, and children’s adjustment in families experiencing intimate partner violence. Journal of Family Violence,23, 243–251. 10.1007/s10896-007-9148-1

[ref40] Jeong, J., Franchett, E.E., Ramos de Oliveira, C.V., Rehmani, K., & Yousafzai, A.K. (2021). Parenting interventions to promote early child development in the first three years of life: A global systematic review and meta-analysis. PLoS Medicine, 18 (5), e1003602. 10.1371/journal.pmed.1003602 33970913 PMC8109838

[ref41] Kline, R.B. (2016). Principles and practice of structural equation modeling. The Guilford Press.

[ref42] Krauss, S., Orth, U., & Robins, R.W. (2020). Family environment and self-esteem development: A longitudinal study from age 10 to 16. Journal of Personality and Social Psychology, 119(2), 457–478. 10.1037/pspp0000263 31535888 PMC7080605

[ref43] Lansford, J.E., Rothenberg, W.A., & Bornstein, M.H. (Eds.). (2021). Parenting across cultures from childhood to adolescence: Development in nine countries (1st ed.). Routledge. 10.4324/9781003027652

[ref44] Lansford, J.E., Sharma, C., Malone, P.S., Woodlief, D., Dodge, K.A., Oburu, P., Pastorelli, C., Skinner, A.T., Sorbring, E., Tapanya, S., Uribe Tirado, L.M., Zelli, A., Al-Hassan, S.M., Alampay, L.P., Bac-chini, D., Bombi, A.S., Bornstein, M.H., Chang, L., Deater-Deckard, K., & Di Giunta, L. (2014). Corporal punishment, maternal warmth, and child adjustment: A longitudinal study in eight countries. Journal of Clinical Child & Adolescent Psychology, 43(4), 670–685. 10.1080/15374416.2014.893518 24885184 PMC4101150

[ref45] Lazarus, R.S., & Folkman, S. (1984). Stress, Appraisal, and Coping. Springer.

[ref46] Leerkes, E.M., & Burney, R.V. (2007). The development of parenting efficacy among new mothers and fathers. Infancy, 12(1), 45–67. 10.1111/j.1532-7078.2007.tb00233.x 33412727

[ref47] Lodi-Smith, J., & DeMarree, K.G. (Eds.). (2018). Self-concept clarity: Perspectives on assessment, research, and applications. Springer. 10.1007/978-3-319-71547-6

[ref48] MacDonald, G., & Leary, M.R. (2005). Why does social exclusion hurt? The relationship between social and physical pain. Psychological Bulletin, 131 (2), 202. 10.1037/0033-2909.131.2.202 15740417

[ref49] Marsh, H.W. (1996). Positive and negative self-esteem: A substantively meaningful distinction or artifactors? Journal of Personality and Social Psychology, 70(4), 810–819. 10.1037/0022-3514.70.4.810 8636900

[ref50] Martín, J.C., Cabrera, E., León, J., & Rodrigo, M.J. (2013). La Escala de Competencia y Resiliencia Parental para madres y padres en contextos de riesgo psicosocial [The Parental Competence and Resilience Scale for Mother and Fathers in At-Risk Psychosocial Contexts]. Anales de Psicología, 29(3), 886–896. 10.6018/analesps.29.3.150981

[ref51] Martorell, G.A., & Bugental, D.B. (2006). Maternal variations in stress reactivity: Implications for harsh parenting practices with very young children. Journal of Family Psychology, 20(4), 641–647. 10.1037/0893-3200.20.4.641 17176199

[ref52] McCarthy, J.D., & Hoge, D.R. (1982). Analysis of age effects in longitudinal studies of adolescent self-esteem. Developmental Psychology, 18(3), 372–379. 10.1037/0012-1649.18.3.372

[ref53] McGinn, L.K., Cukor, D. & Sanderson, W.C. (2005). The relationship between parenting style, cognitive style, and anxiety and depression: Does increased early adversity influence symptom severity through the mediating role of cognitive style? Cognitive Therapy and Research, 29, 219–242. 10.1007/s10608-005-3166-1

[ref54] Mezulis, A.H., Hyde, J.S., & Abramson, L.Y. (2006). The developmental origins of cognitive vulnerability to depression: temperament, parenting, and negative life events in childhood as contributors to negative cognitive style. Developmental Psychology, 42(6), 1012–1025. 10.1037/0012-1649.42.6.1012 17087538

[ref55] Moè, A., & Katz, I. (2018). Brief Research Report: Parents’ Homework Emotions Favor Students’ Homework Emotions Through Self-Efficacy. The Journal of Experimental Education, 86(4), 597–609. 10.1080/00220973.2017.1409180

[ref56] Molano, N., León, E., Jiménez-Morago, J.M., & Camacho, C. (2023). Quality of interactions, children’s psychological adjustment and parental stress in foster families: The mediating role of parental sense of competence. Journal of Child and Family Studies, 32(12), 3601–3611. 10.1007/s10826-023-02578-0

[ref57] Moreira, H., Gouveia, M.J., Carona, C., Silva, N., & Canavarro, M.C. (2015). Maternal attachment and children’s quality of life: The mediating role of self-compassion and parenting stress. Journal of Child and Family Studies, 24, 2332–2344. 10.1007/s10826-014-0036-z

[ref58] Morris, A.S., Silk, J.S., Steinberg, L., Myers, S.S., & Robinson, L.R. (2007). The role of the family context in the development of emotion regulation. Social Development, 16(2), 361–388. 10.1111/j.1467-9507.2007.00389.x 19756175 PMC2743505

[ref59] Muñiz, J., Elosua, P., & Hambleton, R. K. (2013). Directrices para la traducción y adaptación de los tests: Segunda edición [Guidelines for the translation and adaptation of tests: Second edition.]. Psico-thema, 25(2), 151–157. 10.7334/psicothema2013.24 23628527

[ref60] Niermann, C.Y.N., Wagner, P., Ziegeldorf, A., & Wulff, H. (2022). Parents’ and children’s perception of self-efficacy and parental support are related to children’s physical activity: A cross-sectional study of parent–child dyads. Journal of Family Studies, 28(3), 986–1004. 10.1080/13229400.2020.1773901

[ref61] Nunnally, K., & Bernstein, I. (1994). Psychometric theory. McGraw-Hill.

[ref62] Okada, R. (2010). A meta-analytic review of the relation between self-esteem level and self-esteem instability. Personality and Individual Differences, 48(2), 243–246. 10.1016/j.paid.2009.10.012

[ref63] Orth, U., & Robins, R.W. (2014). The development of self-esteem. Current Directions in Psychological Science, 23(5), 381–387. 10.1177/09637214145474

[ref64] Orth, U., Erol, R.Y., & Luciano, E.C. (2018). Development of self-esteem from age 4 to 94 years: A meta-analysis of longitudinal studies. Psychological Bulletin, 144(10), 1045–1080. 10.1037/bul0000161 30010349

[ref65] Oudhof, H., & Robles, E. (2022). Parenthood and childrearing in Mexico: Patterns and trends. In H. Selin (Ed.), Parenting across cultures (Science across cultures: The history of non-Western science, Vol. 12). Springer. 10.1007/978-3-031-15359-4_14

[ref66] Padilla, J.P., Álvarez-Dardet, S.M., & Hidalgo, M.V. (2014). Estrés parental, estrategias de afrontamiento y evaluación del riesgo en madres de familias en riesgo usuarias de los Servicios Sociales [Parental stress, coping strategies and risk assessment in mothers of at-risk families using Social services]. Psychosocial Intervention, 23(1), 25–32. 10.5093/in2014a3

[ref67] Pappa, I., Metallinou, D., Dagla, M., Dragioti, E., Vounatsou, E., Harizopoulou, V.C., & Sarantaki, A. (2024). Breaking the silence: Exploring fathers’ perspectives on perinatal mental health and navigating fatherhood. Preprints. 10.20944/preprints202411.0997.v1

[ref68] Peng, B., Hu, N., Yu, H., Xiao, H., & Luo, J. (2021). Parenting style and adolescent mental health: The chain mediating role of self-esteem and psychological inflexibility. Frontiers in Psychology, 12, 738170. 10.3389/fpsyg.2021.738170 34721210 PMC8548717

[ref69] Pinquart, M. (2017). Associations of parenting dimensions and styles with internalizing symptoms in children and adolescents: A meta-analysis. Marriage & Family Review, 53(7), 613–640. 10.1080/01494929.2016.1247761

[ref70] Pinquart, M., & Gerke, D.-C. (2019). Associations of parenting styles with self-esteem in children and adolescents: A meta-analysis. Journal of Child and Family Studies, 28, 2017–2035. 10.1007/s10826-019-01417-5

[ref71] Pinquart, M., & Nguyen, V.A. (2025). Cultural similarities and differences in the association of parental autonomy support with internalizing and externalizing problems: A meta-analysis. Journal of Child and Family Studies, 35, 1141–1152. 10.1007/s10826-026-03281-6

[ref72] Pisterman, S., Firestone, P., McGrath, P., Goodman, J.T., Webster, I., Mallory, R., & Coffin, B. (1992). The effects of parent training on parenting stress and sense of competence. Canadian Journal of Behavioural Science/Revue canadienne des sciences du comportement, 24(1), 41–58. 10.1037/h0078699

[ref73] Ponnet, K., Mortelmans, D., Wouters, E., Van Leeuwen, K., Bastaits, K., & Pasteels, I. (2013). Parenting stress and marital relationship as determinants of mothers’ and fathers’ parenting. Personal Relationships, 20(2), 259–276. 10.1111/j.1475-6811.2012.01404.x

[ref74] Prasana, M., & Sam, K.M. (2024). Influence of perceived parenting style on self-esteem and stress among adolescents. The International Journal of Indian Psychology, 12(3), 1521–1535.

[ref75] Putnick, D.L., Bornstein, M.H., Hendricks, C., Painter, K.M., Suwalsky, J.T.D., & Collins, W.A. (2008). Parenting stress, perceived parenting behaviors, and adolescent self-concept in European American families. Journal of Family Psychology, 22(5), 752–762. 10.1037/a0013177 18855511 PMC2575655

[ref76] Rodrigo, M.J., Martín, J.C., Cabrera, E., & Máiquez, M.L. (2009). Las competencias parentales en contextos de riesgo psicosocial [Parental competences in contexts of psychosocial risk]. Intervención Psicosocial, 18(2), 113–120. 10.5093/in2009v18n2a2

[ref77] Rodriguez-Jenkins, J., & Marcenko, M.O. (2014). Parenting stress among child welfare involved families: Differences by child placement. Children and Youth Services Review, 46, 19–27. 10.1016/j.childyouth.2014.07.024 26170514 PMC4498480

[ref78] Rohmalimna, A., Yeau, O., & Sie, P. (2022). The role of parental parenting in the formation of the child’s self-concept. World Psychology, 1(2), 36–45. 10.55849/wp.v1i2.99

[ref79] Rosenberg, M. (1965). Society and the adolescent self-image. Princeton University Press. 10.1515/9781400876136

[ref80] Rosenberg, M., Schooler, C., & Schoenbach, C. (1989). Self-Esteem and Adolescent Problems: Modeling Reciprocal Effects. American Sociological Review, 54(6), 1004–1018. 10.2307/2095720

[ref81] Ruiz, M.A., Pardo, A., & Martín, R.S. (2010). Modelos de ecuaciones estructurales [Models of structural equations]. Papeles Del Psicologo, 31(1), 34–45. https://www.papelesdelpsicologo.es/pdf/1794.pdf

[ref82] Rusu, P.P., Candel, O.S., Bogdan, I., Ilciuc, C., Ursu, A., & Podina, I.R. (2025). Parental stress and wellbeing: A meta-analysis. Clinical Child and Family Psychology Review, 28, 255–274. 10.1007/s10567-025-00515-9 40057656 PMC12162691

[ref83] Ryff, C.D. (2014). Psychological well-being revisited: Advances in the science and practice of eudai-monia. Psychotherapy and Psychosomatics, 83, 10–28. 10.1159/000353263 24281296 PMC4241300

[ref84] Sahithya, B.R., & Raman, V. (2020). Parenting styles, temperament, and anxiety in children: Preliminary findings in the Indian population. Open Journal of Psychiatry & Allied Sciences, 11(1), 19–25. 10.5958/2394-2061.2020.00005.1

[ref85] Sahithya, B.R., Rithvik, K., & Roopesh, B. (2020). Perceived stress, parental stress, and parenting during COVID-19 Lockdown: A preliminary study. Journal of Indian Association for Child and Adolescent Mental Health, 16(4), 44–63. 10.1177/0973134220200404

[ref86] Shen, L.A., Ren-Fang, C., & Wang, W.C. (2025). How parents of young children perceive the value of participating in parent-child physical activities. International Journal of Research in Business & Social Science, 14(7), 463–472. 10.20525/ijrbs.v14i7.4452

[ref87] Smith, T.D., & McMillan, B.F. (2001, February 1–3). A primer of model fit indices in structural equation modeling [Conference session]. Annual Meeting of the Southwest Educational Research Association, New Orleans, LA, United States. http://eric.ed.gov/ERICWebPortal/recordDetail?accno=ED449231

[ref88] Stommel, M., Wang, S., Given, C.W., & Given, B. (1992). Focus on psychometrics confirmatory factor analysis (CFA) as a method to assess measurement equivalence. Research in Nursing & Health, 15(5), 399–405. 10.1002/nur.4770150508 1529124

[ref89] Sugiarti, R., Erlangga, E., Purwaningtyastuti, P., & Suhariadi, F. (2021). The influence of parenting and friendship on self-esteem in adolescents. Open Access Macedonian Journal of Medical Sciences, 9, 1307–1315. 10.3889/oamjms.2021.6881

[ref90] Swerdlik, M.E., & Cohen, R. (2006). Pruebas y evaluación psicológicas. Introducción a las pruebas y a la medición [Psychological tests and evaluation. Introduction to testing and measurement]. McGraw-Hill.

[ref91] Taylor, S.E., & Brown, J.D. (1988). Illusion and well-being: a social psychological perspective on mental health. Psychological Bulletin, 103(2), 193–210. 10.1037/0033-2909.103.2.193 3283814

[ref92] Tehrani, H.D., Yamini, S., & Vazsonyi, A.T. (2025). The links between parenting, self-esteem, and depressive symptoms: A meta-analysis. Journal of Adolescence, 97(2), 315–332. 10.1002/jad.12435 39472151

[ref93] Tipton, E. (2017). Implications of small samples for generalization: Adjustments and rules of thumb. Evaluation Review, 41(5), 472–505. 10.1177/0193841X16655665 27402612

[ref94] Triola, M. (2009). Estadística [Statistics] (10ma ed.). Pearson Educación.

[ref95] Wekerle, C., Miller, A., Wolfe, D., & Spindel, C. (2007). Maltrato infantil [Child maltreatment]. Manual Moderno.

[ref96] Wylie, R.C. (1974). The self-concept: Theory and research on selected topics (Vol. 2). University of Nebraska Press.

[ref97] Yong, A.G., & Pearce, S. (2013). A beginner’s guide to factor analysis: Focusing on Exploratory Factor Analysis. Tutorials in Quantitative Methods for Psychology, 9(2), 79–94. 10.20982/tqmp.09.2.p079

